# Characterization of porphobilinogen deaminase mutants reveals that arginine-173 is crucial for polypyrrole elongation mechanism

**DOI:** 10.1016/j.isci.2021.102152

**Published:** 2021-02-06

**Authors:** Helene J. Bustad, Juha P. Kallio, Mikko Laitaoja, Karen Toska, Inari Kursula, Aurora Martinez, Janne Jänis

**Affiliations:** 1 Department of Biomedicine, University of Bergen, Jonas Lies vei 91, 5009 Bergen, Norway; 2 Department of Chemistry, University of Eastern Finland, 80130 Joensuu, Finland; 3 Norwegian Porphyria Centre (NAPOS), Department for Medical Biochemistry and Pharmacology, Haukeland University Hospital, 5021 Bergen, Norway; 4 Faculty of Biochemistry and Molecular Medicine, University of Oulu, 90570 Oulu, Finland

**Keywords:** Biological Sciences, Biochemistry, Structural Biology, Proteomics

## Abstract

Porphobilinogen deaminase (PBGD), the third enzyme in the heme biosynthesis, catalyzes the sequential coupling of four porphobilinogen (PBG) molecules into a heme precursor. Mutations in PBGD are associated with acute intermittent porphyria (AIP), a rare metabolic disorder. We used Fourier transform ion cyclotron resonance mass spectrometry (FT-ICR MS) to demonstrate that wild-type PBGD and AIP-associated mutant R167W both existed as holoenzymes (E_holo_) covalently attached to the dipyrromethane cofactor, and three intermediate complexes, ES, ES_2_, and ES_3_, where S represents PBG. In contrast, only ES_2_ was detected in AIP-associated mutant R173W, indicating that the formation of ES_3_ is inhibited. The R173W crystal structure in the ES_2_-state revealed major rearrangements of the loops around the active site, compared to wild-type PBGD in the E_holo_-state. These results contribute to elucidating the structural pathogenesis of two common AIP-associated mutations and reveal the important structural role of Arg173 in the polypyrrole elongation mechanism.

## Highlights

• FT-ICR MS provides molecular information of enzyme-intermediate complexes of PBGD
• FT-ICR MS is optimal to elucidate the catalytic defect in AIP-associated PBGD mutants
• The structure of R173W-PBGD shows a disease mutant trapped in a reaction intermediate
• Arg173 is crucial for the polypyrrole elongation beyond the ES_2_ intermediate state

## Graphical abstract

**Figure:**
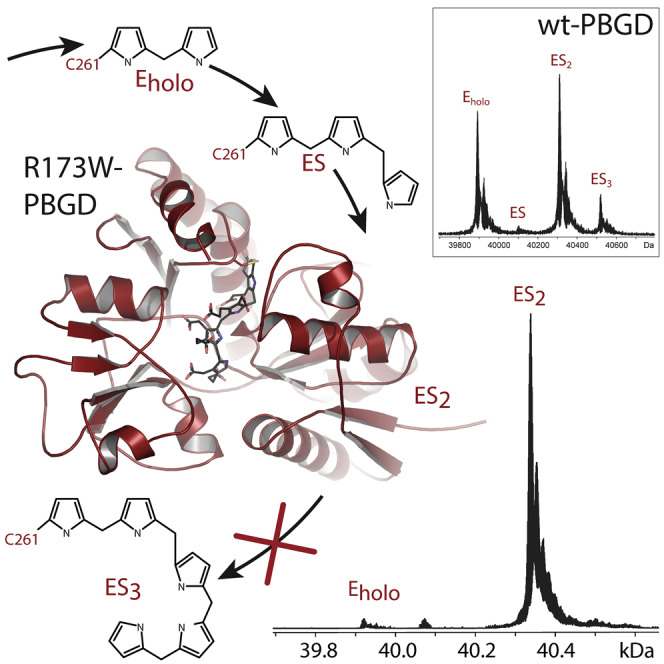

## Introduction

Porphobilinogen deaminase (PBGD; EC 2.5.1.61), also known as hydroxymethylbilane synthase (HMBS), is the third enzyme in the heme biosynthetic pathway. Heme is an important biomolecule that participates in many essential functions in humans, in particular, oxygen transport in blood. PBGD catalyzes four consecutive reactions to convert porphobilinogen (PBG) into hydroxymethylbilane (HMB), a linear tetrapyrrole heme precursor. Mutations in the *HMBS* gene are associated with a genetic metabolic disorder known as acute intermittent porphyria (AIP), giving reduced heme production and severe metabolic and neurological symptoms (Bonkovsky et al., 2019). Missense mutations constitute ∼32% of the more than 500 known mutations of human PBGD (hPBGD), and cause destabilization of the enzyme and/or a direct effect on catalysis (Chen et al., 2019; Scott et al., 1988). PBGD is expressed in a tissue-specific manner, with two isoforms produced by different promotor usage and alternative splicing, with the ubiquitously expressed housekeeping PBGD containing 17 extra residues in the N-terminus compared to the erythroid-specific isoform (Grandchamp et al., 1987). However, the catalytic activity of these two isoforms is similar, and the extra N-terminal region has no known function (Brons-Poulsen et al., 2005).

**Figure 1:**
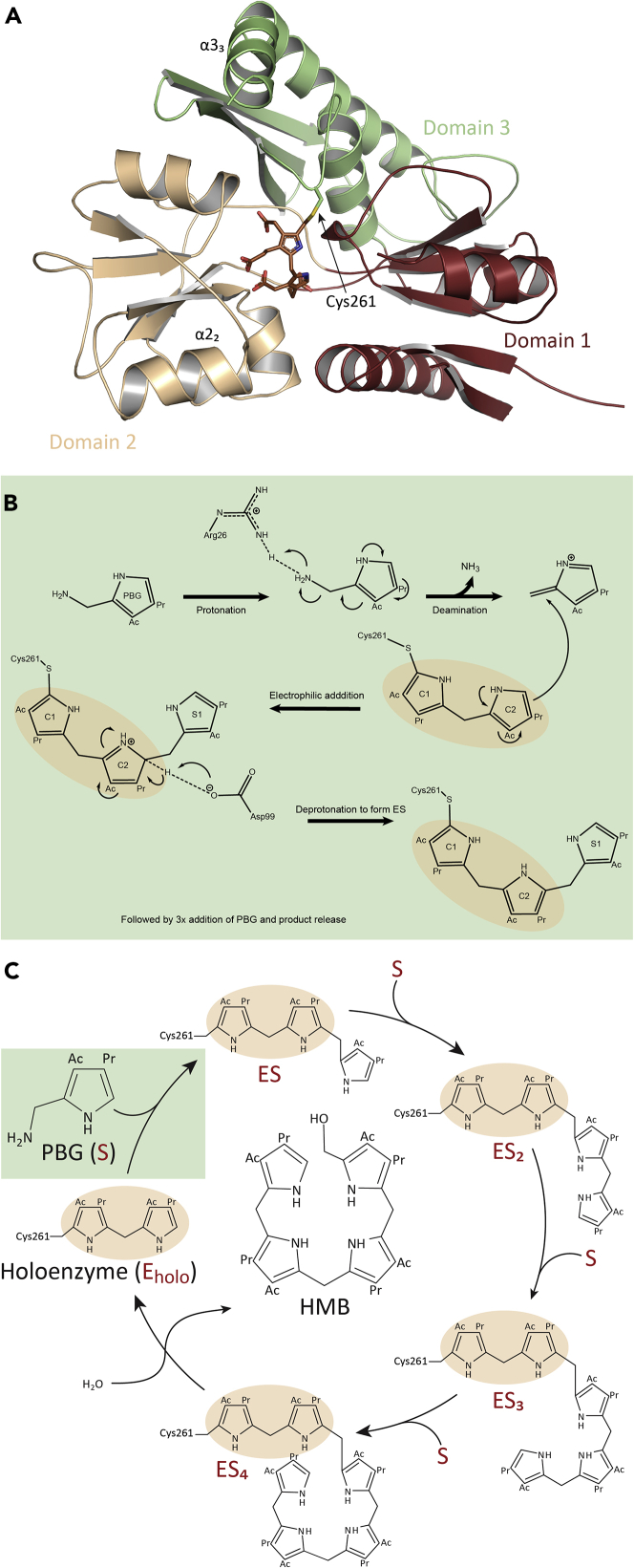
Crystal structure of hPBGD wt-E_holo_ and schematic representation of the polypyrrole elongation mechanism (A) Overall representation of the crystal structure of wt-hPBGD (PDB: 7AAJ), providing the expected holoenzyme (E_holo_) with three separate domains (domain 1 is presented in red, domain 2 in light brown, and domain 3 in green). The bound dipyrromethane (DPM) cofactor is shown in a brown stick representation. (B) Schematic representation of the general understanding of the mechanism of a single step in the polypyrrole elongation. (C) The polypyrrole elongation catalyzed by PBGD. PBGD with attached DPM (E_holo_) subsequently binds four porphobilinogen (PBG) substrates (S), and generates the enzyme intermediates ES, ES_2_, ES_3_, and ES_4_. The linear product, hydroxymethylbilane (HMB), is released by hydrolysis and cyclized by the next enzyme in the heme biosynthesis. The sidechains of the substrates, acetate (CH_2_CO_2_H) and propionate (CH_2_CH_2_CO_2_H) are denoted Ac and Pr, respectively. Figure 1C is modified from (Jordan and Woodcock, 1991).

A wealth of structural and functional information is available for both wild-type (wt) and mutant PBGDs, including several three-dimensional structures as well as kinetic, biophysical, and computational data (Awan et al., 1997; Bung et al., 2019; Bustad et al., 2013; Hadener et al., 1999; Louie et al., 1992; Niemann et al., 1994; Pluta et al., 2018; Roberts et al., 2013; Shoolingin-Jordan et al., 1996; Song et al., 2009). The crystal structures of wt-PBGD have so far been solved for human (PDB: 3ECR, 5M7F (Pluta et al., 2018; Song et al., 2009)), *Escherichia coli* (PDB: 1PDA (Louie et al., 1992)), *Arabidopsis thaliana* (PDB: 4HTG (Roberts et al., 2013)), *Bacillus megaterium* (PDB: 4MLV (Azim et al., 2014)) and *Vibrio cholera* (PDB: 5H6O (Uchida et al., 2018)) enzymes, showing the same basic topology with three separate domains (Figure 1A). A deep cleft within the active site connects domains 1 and 2 (residues 1–114 and 120–212, respectively, in hPBGD) by several interactions that stabilize the overall structure. The holoenzyme (E_holo_) carries a dipyrromethane (DPM) cofactor, which is covalently linked to a conserved cysteine residue (Cys261 in hPBGD) through a thioether bond (Song et al., 2009). Domain 3 (residues 241–361) contains a loop that includes the active site Cys261. DPM derives from two PBG molecules, and functions as an anchor for the sequential coupling of additional four PBG molecules by deamination (Figure 1B) (Layer et al., 2010). When the polypyrrole chain elongation is complete, HMB is released by hydrolysis of the thioether bond, leaving DPM behind, after which the cycle starts again (Figure 1C). As a part of the head-to-tail polymerization process, several covalent enzyme-substrate intermediates are formed, first described in the early 1980s (Anderson and Desnick, 1980; Berry et al., 1981; Jordan and Berry, 1981), which are usually denoted as ES, ES_2_, ES_3_, and ES_4_, where E represents E_holo_, and S represents the reacted PBG molecule.

The exact mechanism of the polypyrrole formation is still not completely understood. Recently, Pluta et al. published crystal structures of the wt-hPBGD E_holo_ and its ES_2_ intermediate (PDB: 5M7F and 5M6R, respectively (Pluta et al., 2018)). These structures allowed the authors to propose a reaction mechanism for PBGD, highlighting the importance of the flexible loop Leu257–Val263 that gives room for the elongation from E_holo_ to ES_2_. This is contrary to recent molecular dynamics (MD) simulations complemented with site-directed mutagenesis where very little structural rearrangements upon polypyrrole formation were associated with catalysis and the authors suggested that only a few specific residues, namely Asp99 and Arg26 (Figure 1B), would be responsible for the elongation process (Bung et al., 2014, 2018, 2019).

In this work, we analyzed wt-hPBGD and two recurrent AIP-associated mutants using high-resolution electrospray ionization Fourier transform ion cyclotron resonance mass spectrometry (ESI FT-ICR MS) and X-ray crystallography. The mutants, R167W and R173W, have different mechanistic effects, but are both associated with a severe AIP phenotype (Andersson et al., 2000; Bustad et al., 2013; Mustajoki et al., 2000). R167W represents a catalytically impaired mutant owing to high *K*_m_ for PBG and low *V*_max_ leading to slower polypyrrole elongation (Bustad et al., 2013; Fu et al., 2019; Solis et al., 2004). R173W, on the other hand, is catalytically deleterious with an activity of 0.6% relative to wt and indeterminable *K*_m_ and *V*_max_, together with an obstructed substrate elongation (Bustad et al., 2013; Fu et al., 2019). Our results indicate that Arg173 is essential for the formation of the ES_3_ intermediate, with an important structural role that is relevant for its catalytic contribution. Considering this, we provide unique molecular details on the reaction intermediates for wt-hPBGD and the AIP-associated mutants and shed light on the catalytic and pathogenic mechanisms.

## Results

### Characterization of enzyme intermediates in the wild-type hPBGD

**Figure 2:**
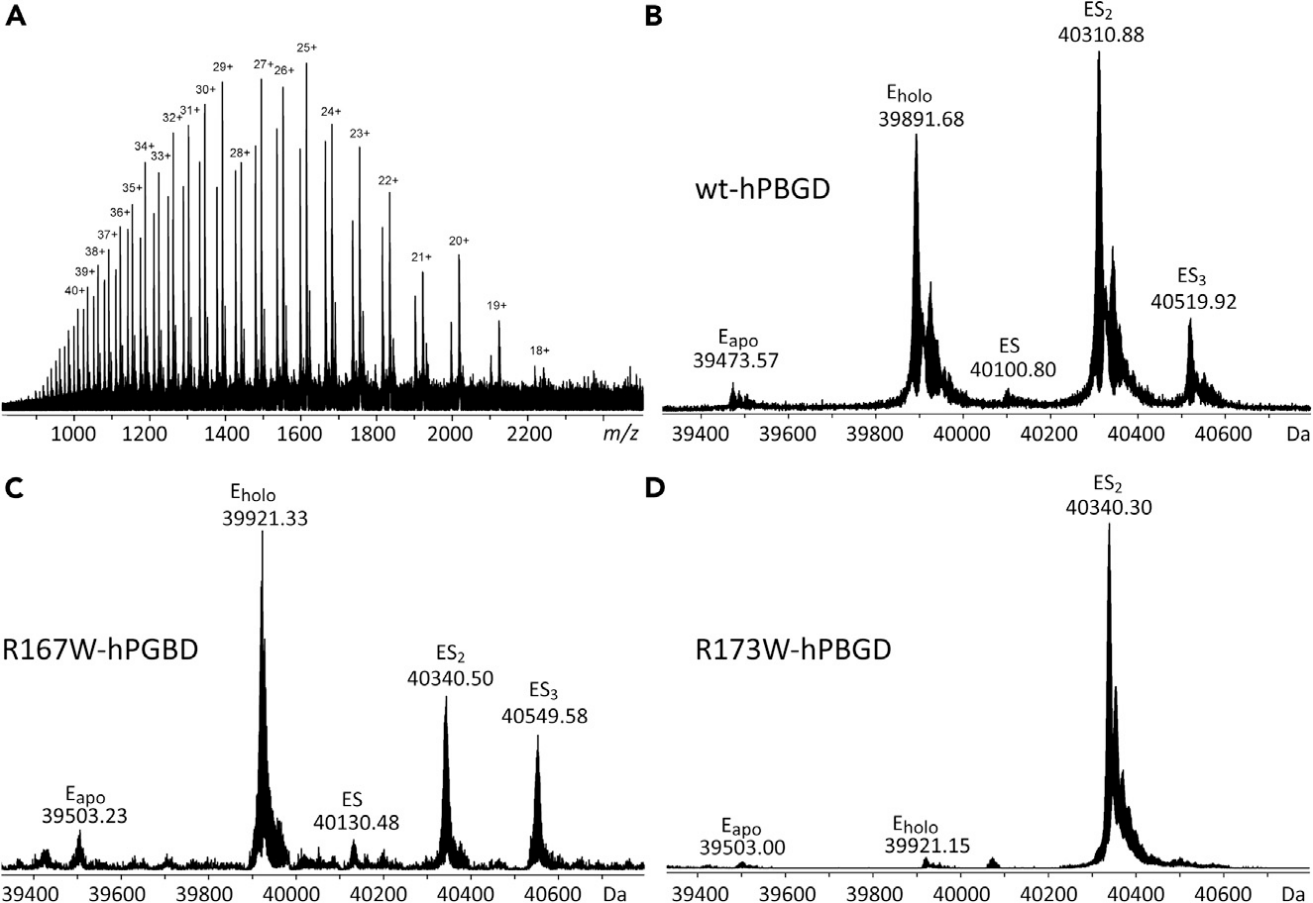
High-resolution mass spectrometry of hPBGD enzymes The ESI FT-ICR mass spectra were measured at denaturing conditions with 5 μM protein. (A) Broadband mass spectrum of wt-hPBGD with numbers denoting different protein ion-charge states. A wide charge state distribution from 18 + to 45+ is consistent with the protein being fully unfolded. (B) Charge-deconvoluted mass spectrum showing peaks representing different enzyme-intermediates in wt-hPBGD. (C) and (D) Charge-deconvoluted mass spectra of the hPBGD mutants R167W and R173W, respectively. The peaks representing different enzyme-intermediates are assigned. See also Figures S1–S3, and Tables S1 and S2.

The recombinantly expressed and purified hPBGD typically consists of a heterogeneous mixture of enzyme-intermediates (Bustad et al., 2013; Shoolingin-Jordan et al., 2003), and isolation of each intermediate is not customarily performed. In the ESI-FT-ICR mass spectra of wt-hPBGD, all the expected enzyme intermediates, i.e., E_holo_, ES, ES_2_, and ES_3_, were detected (Figures 2A and 2B). In addition, a small amount of the apoenzyme (E_apo_) was also observed, which is surprising since E_apo_ is expected to be highly unstable and has not been observed in earlier studies of PBGD kinetic intermediates. However, ESI-FT-ICR MS is a much more sensitive detection technique than most other biophysical methods previously applied. The experimentally determined masses of the intermediates perfectly matched with the theoretical masses considering covalently linked reaction intermediates. The greatest abundance was observed for ES_2_, followed by E_holo_, ES_3_, and ES complexes (Figure 2B), corroborating that ES_2_ is kinetically the most stable reaction intermediate. As expected, ES_4_ was not detected at all, as this intermediate is short-lived and is rapidly hydrolyzed into the linear HMB product (Warren and Jordan, 1988). This observed distribution is consistent with results from earlier studies (Bustad et al., 2013; Niemann et al., 1994; Shoolingin-Jordan et al., 2003), demonstrating the ability of ESI-FT-ICR MS to separate and directly identify different co-existing enzyme-substrate intermediates through intact protein mass analysis. In addition, the mass accuracy was high enough to directly distinguish between the reduced (DPM) and the oxidized (dipyrromethene) form of the cofactor (i.e., 2 Da mass difference in a 40 kDa protein), and our results conclusively indicated that the DPM cofactor existed exclusively in its reduced DPM form (Figure S1). There was no evidence of further cofactor oxidation to a dipyrromethanone (+16 Da) form, which has been observed in the crystal structure of *B. megaterium* PBGD and suggested to be responsible for the pink color of the enzyme in solution (Azim et al., 2014).

We also confirmed that the substrate binding exclusively occurs through the covalent linkage to Cys261. The tryptic digestion of wt-hPBGD resulted in nearly 100% sequence coverage with 58 identified specific tryptic peptides (Table S1 and Figure S2). The peptide 226–272 was only identified when the tetrapyrrole (corresponding to ES_2_) was included as the variable modification in the peptide fingerprinting.

Furthermore, a comparison of the ESI FT-ICR mass spectra of wt-hPBGD in denaturing (Figure 2B) and native conditions (10 mM ammonium acetate pH 6.9; Figure S1) showed similar enzyme-intermediates, thus, implying that all enzyme-substrate complexes are covalent in nature. The only difference between the spectra was that weak signals were detected at *m/z* 3800–4500 at native conditions, possibly representing a very low proportion of a noncovalent protein dimer (Figure S1).

### The relative amounts of the reaction intermediates are different for the AIP-associated hPBGD mutants compared to the wild-type enzyme

Two active site hPBGD mutants, R167W and R173W, both showing catalytic dysfunction and a high association with AIP (Bustad et al., 2013), were selected for mass spectrometric analysis. For R167W, the observed enzyme intermediates by ESI FT-ICR MS in denaturing conditions were like wt-hPBGD (Figures 2B and 2C). However, a much higher relative abundance was seen for E_holo_ as compared to the other reaction intermediates, suggesting that the mutation R167W causes a perturbed binding of the first PBG molecule to the E_holo_ and decreasing the rate of HMB synthesis.

In contrast, only a single reaction intermediate was observed for R173W, with a mass corresponding to ES_2_ (Figure 2D). This result is consistent with the previous native PAGE analysis of this mutant with a single protein band, and a mild conformational defect as seen by thermal circular dichroism spectroscopy and differential scanning fluorimetry (Bustad et al., 2013). This suggests that productive binding of a third substrate molecule is inhibited when Arg173 is mutated to tryptophan, leading to the accumulation of the ES_2_ intermediate without turnover, in agreement with the more severe catalytic dysfunction (<1% residual activity) and a more severe AIP phenotype for the R173W than for the R167W mutant (Fu et al., 2019). Additional trypsin digestion experiments verified that the cofactor or the growing pyrrole chain were bound exclusively to Cys261 also in R173W-hPBGD (Table S2 and Figure S3).

### High-resolution crystal structures provide important insights into the catalytic mechanism

To obtain further structural insight into the catalytic mechanism we crystallized wt-hPBGD and the AIP-associated mutant R173W. The three-dimensional structures were determined to 1.8 (PBD: 7AAJ) and 1.7 Å resolution (PBD: 7AAK), respectively (Table 1). The overall three-domain structure of both proteins, as well as the active site architecture of wt-hPBGD, are very similar to those of the previously published structure, with an RMSD of 0.256 Å between monomers of our wt-hPBGD and PDB: 3ECR (Figure 1A). Furthermore, both our structures contain two monomers in the asymmetric unit without indication of dimerization as also supported by the mass spectrometry results showing that except for minor dimeric forms, E_holo_ and the other enzyme-intermediates are monomeric. Our crystallization trials for the R167W mutant were unsuccessful. However, a previously published structure of the R167Q mutant (PDB: 3EQ1 (Gill et al., 2009)) also represents the E_holo_-state only, as the wt-hPBGD.

**Figure 3:**
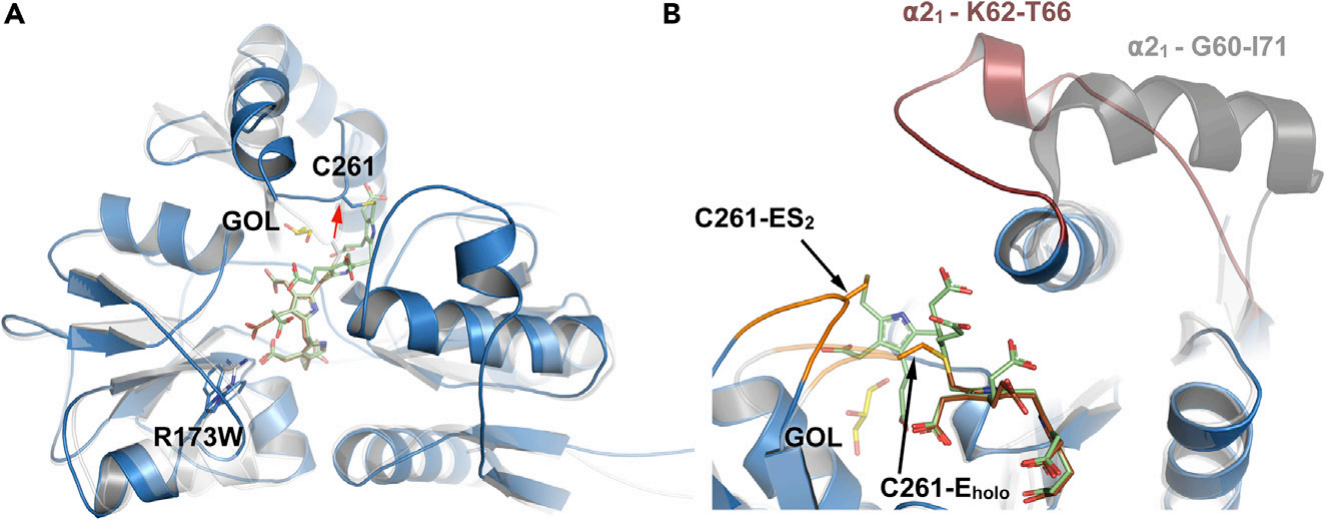
The crystal structure of hPBGD wt-E_holo_ and R173W-ES_2_ (A) Overall cartoon representation of wt-E_holo_ (gray; PDB: 7AAJ) and mutant R173W-ES_2_ (blue; PDB: 7AAK) superimposed. DPM cofactor with C1 and C2 units of wt-E_holo_ is shown in brown and elongation product of R173W-ES_2_ including S1 and S2 units is shown in green. The mutated residue studied here, R173W, is shown as sticks. The cofactor-binding loop and cofactor are rearranged (red arrow) in the structure, allowing incoming substrate pyrroles (S1 and S2) substitute C1 and C2 at equal positions as in the E_holo_. (B) The active-site loop (residues 57–74) orientation in wt-ES_2_ (PDB: 5M6R; dark gray (Pluta et al., 2018)) is compared to the loop orientation in R173W-ES_2_ (red). Formed ⍺2_1_ helixes are labeled. Close-up also shows the movement of the cofactor-binding loop (orange; residues 257–263), and the elongation product in the R173W mutant. The position of cofactor-binding Cys261 has been indicated with labels C261-E_holo_ and C261-ES_2_ for wt-E_holo_ and R173W-ES_2_ structures, respectively. Glycerol (GOL) partially filling the solvent cavity under the cofactor-binding loop in the R173W-ES_2_ structure is shown as sticks (yellow). See also Figures S4 and S5.

**Figure 4:**
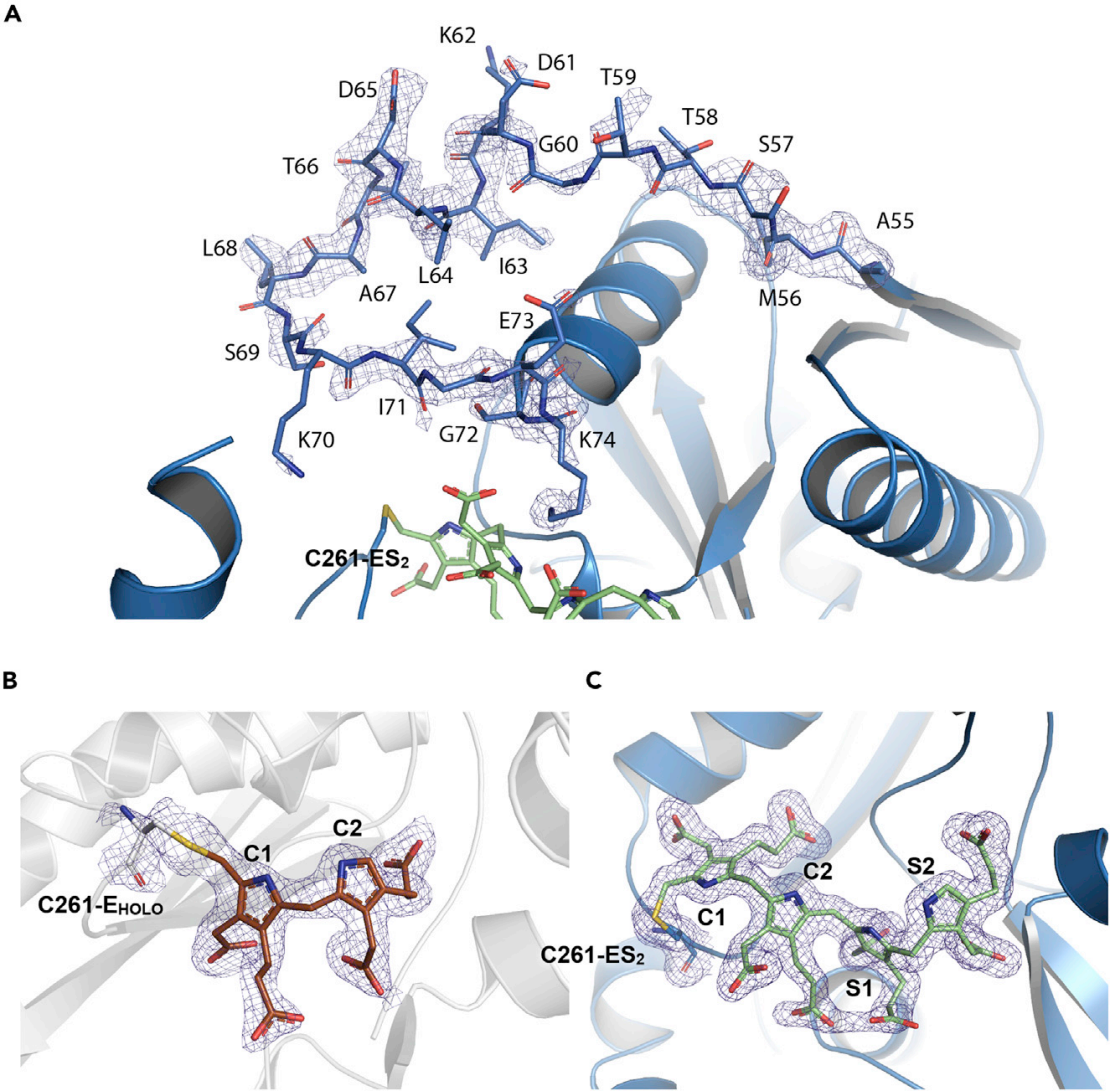
Electron density for the structural features Calculated 2mFo-DFc-electron density for (A) the active-site loop in R173W-ES_2_, (B) bound cofactor in wt-E_holo_ and (C) the polypyrrole chain in R173W-ES_2_. Electron density is contoured with sigma level of 1.0.

**Figure 5:**
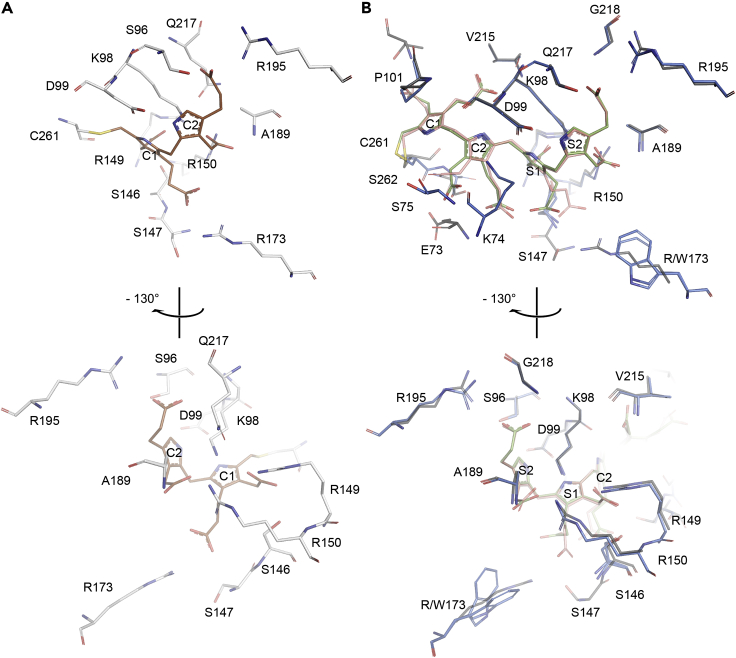
Stick representation of the active site The image shows active site interactions of (A) our wt-E_holo_ (side chains gray and substrate brown), and (B) R173W-ES_2_ (side chains blue and substrate green) superimposed with wt-ES_2_ (PDB: 5M6R; side chains dark gray and substrate pink (Pluta et al., 2018)). Incoming PBG units in the ES_2_ intermediates substitute C1 and C2 at equal positions as in the E_holo_. Interactions are described in detail in Table 2.

**Figure 6:**
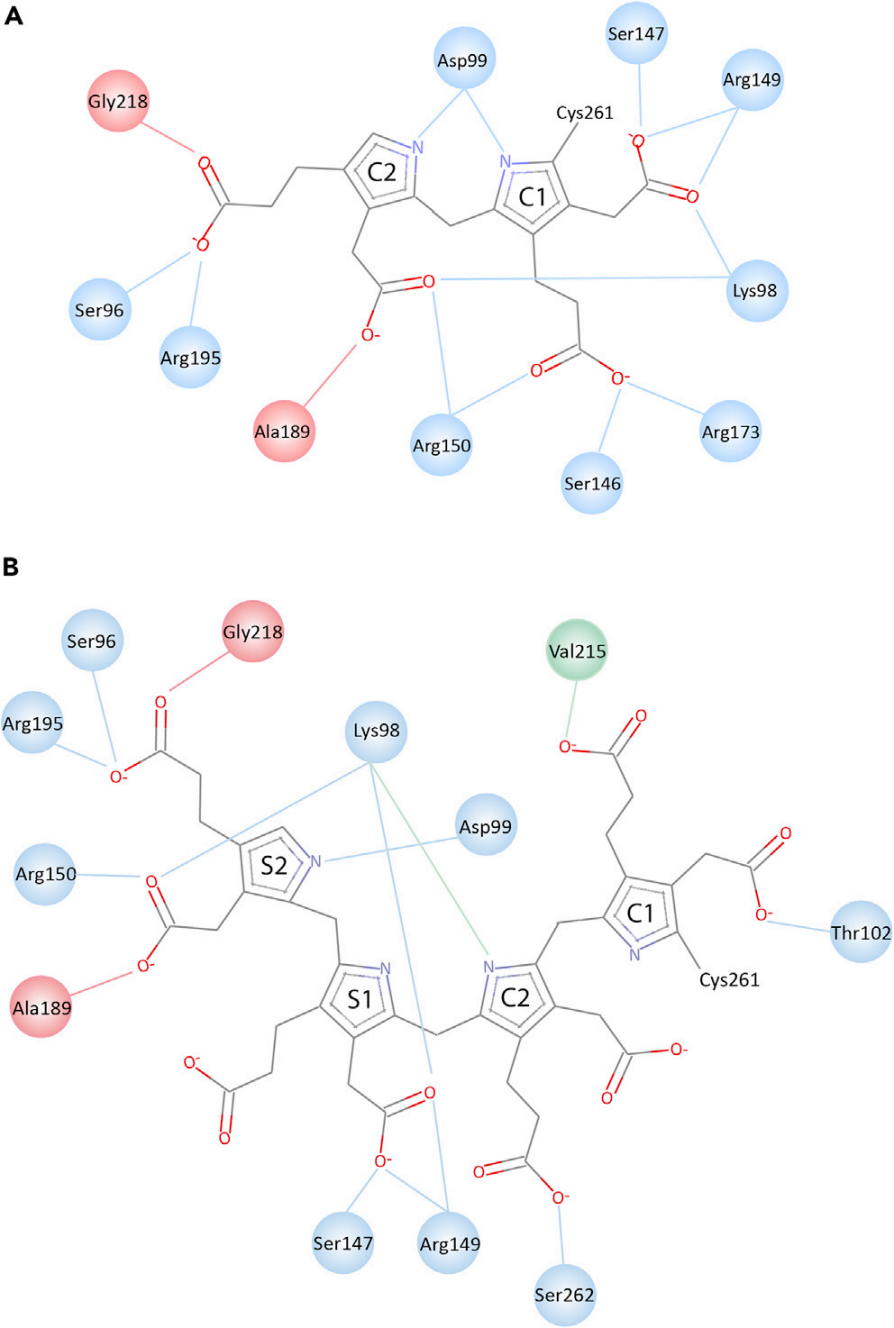
Schematic view of the interactions between the pyrrole rings and the hPBGD protein in the crystal structures of wt-E_holo_ and R173W-ES_2_ mutant (A) The hydrogen bond interactions of the DPM cofactor with the wt-hPBGD residues in the active site. (B) The interactions between the pyrrole chain intermediate as seen in the crystal structure of the R173W-hPBGD mutant. Side chain H-bond interactions (blue), H-bond interactions to main chain carbonyl oxygen (green) and H-bond interactions to nitrogen (red).

In agreement with the ESI FT-ICR MS analyses of wt-hPBGD, a fully reduced covalently attached DPM occupies the active site cleft, showing a ∼120° angle between the pyrrole rings, instead of the coplanar conformation of oxidized DPM (Azim et al., 2014). The crystal structure only represents the E_holo_-state and is denoted as wt-E_holo_ hereafter. The binding mode (Figures 3 and 4) and interactions (Figures 5A and 6A) of DPM are the same as in the previously published structure (PDB: 3ECR). Electron density is observed for the two neighboring residues of Cys261, i.e., Gly260 and Gly259, which are also visible in another recent structure of wt-hPBGD (PDB: 5M7F) but not in the earlier structure (PDB: 3ECR). The variability of the electron density quality as well as elevated B-factors indicate a dynamic nature of the residues Leu257–Val263 that constitute the cofactor-binding loop (Figure 3B). Electron density is missing for the first 18 residues at the N-terminus and for the active-site loop residues Ser57–Lys74. Unfortunately, surface exposed residues in flexible sidechains, including Arg167, are not described in the electron density maps. This prevented us to draw further conclusions on the possible catalytic role of this residue.

The structure of the mutant R173W-hPBGD agrees with the ESI FT-ICR MS analysis, representing only ES_2_ in its reduced form (denoted R173W-ES_2_) and thus revealing for the first time an AIP-associated mutant trapped in a reaction intermediate state. Electron density for the N-terminus was again missing, and the main chain atoms of the active-site loop Ser57–Lys74 could be built only in subunit A (Figure 4A). Within this loop, residues Lys62–Thr66 form a short α-helix (Figure 3B) shown with individual residues in the electron density map in Figure 4A. Crystal packing prevents the loop in the other subunit, B, to adopt the same conformation as in subunit A, however, only traces of the electron density for the loop in subunit B can be seen and was thus not built. Trp173 has different side chain conformations in the two subunits and has two alternative conformations in subunit A (Figure S4). In addition, Ser146 has alternative conformations and seems to move in concert with Trp173 (Figure S4). We also discovered rearrangement in the helix α2_2_ (residues 170–179; Figure S4) in subunit B. This rearrangement allows Trp173 to adopt a completely different conformation than seen in subunit A. From this point forward, we will only discuss the structure of subunit A, unless otherwise stated.

The additional pyrrole rings seen in the R173W-ES_2_ structure are denoted S1 and S2, in addition to the C1 and C2 rings of the original DPM cofactor (Figures 4, 5, and 6). The incorporation of S1 and S2 in R173W-ES_2_ causes a major rearrangement in the cofactor-binding loop Leu257–Val263 including Cys261 to which the reaction intermediate is covalently bound. In contrast to wt-E_holo_, the active-site loop Ser57–Lys74 in R173W-ES_2_ is reoriented toward the C-terminal helix, α3_3,_ allowing the accommodation of the additional pyrrole rings in the active site cleft between the domains 1 and 2 (Figure 3). The created cavity is not filled by the relocated C1 and C2 rings, and in the structure, a glycerol molecule occupies the space between C1 and the protein core (Figure 3). Because of this rearrangement, the new pyrrole rings S1 and S2 take the original places of the C1 and C2 rings of the DPM cofactor in the wt-E_holo_ structure (Figures 3B and 5).

### Structural comparison between R173W-ES_2_, wt-E_holo_ and wt-ES_2_

All three domains participate in the formation of the interaction network around the cofactor in E_holo_ or the four pyrrole rings in ES_2_ in the active site. These interactions are described in detail in Table 2, and Figures 5 and 6. It is noticeable that the interactions change for C1 and C2 when S1 and S2 subsequently are incorporated in the R173W-ES_2_ structure. Thus, C1 interacts with Thr102 and Val215 (main chain), and C2 interacts with Lys98 (main chain) and Ser262 (Figures 5 and 6). S1, which in R173W-ES_2_ occupies the original position of C1 in E_holo_, interacts with Ser147 and Arg149 through the acetate side chain. The propionate side chain of S1 does not make hydrogen bonds to any atoms in the R173W-ES_2_ structure. S2 occupies the equivalent position of C2 in E_holo_, where the acetate side chain interacts with Arg98, Arg150 and Ala189 and the propionate side chain creates hydrogen bonds with Ser96, Arg195, and Gly218. Lys98 forms salt bridges with both acetate side chains of rings S1 and S2. Interestingly Asp99 interacts with both pyrrole N atoms of C1 and C2 in wt-E_holo_, whereas in the R173W-ES_2_ there is only an interaction to pyrrole N in S1 (Table 2 and Figures 5 and 6).

**Table 1:** Data collection and refinement statistics

	wt-E_holo_	R173W-ES_2_
**Data collection**		
PDB ID	7AAJ	7AAK
Resolution range	65.08–1.8 (1.864–1.8)	59.1–1.7 (1.761–1.7)
Space group	P 2_1_ 2_1_ 2_1_	P 2_1_ 2_1_ 2_1_
Unit cell	81.2 84.6 108.9 90 90 90	81.2 86.1 107.4 90 90 90
Total reflections	528551 (52,834)	551220 (55,773)
Unique reflections	69,757 (6877)	83,105 (8199)
Multiplicity	7.6 (7.7)	6.6 (6.8)
Completeness (%)	98.9 (97.3)	99.47 (99.22)
Mean I/sigma(I)	10.24 (0.52)	15.48 (1.62)
Wilson B-factor	34.2	25.41
R-merge	0.095 (3.76)	0.068(1.56)
R-meas	0.102 (4.02)	0.074 (1.69)
CC1/2	0.99 (0.54)	1 (0.73)

**Refinement**		
Reflections used in refinement	69,336 (6735)	82,994 (8182)
Reflections used for R-free	3510 (280)	4022 (398)
R_work_	24.9 (62.2)	18.3(34.8)
R_free_	29.4 (65.3)	21.2 (35.8)
Number of non-hydrogen atoms	5239	5918
Macromolecules	5000	5267
Ligands	78	144
Solvent	161	507
Protein residues	647	669
RMSD (bonds)	0.007	0.007
RMSD (angles)	0.8	0.8

**Validation**		
Ramachandran favored (%)	96.8	98.2
Ramachandran allowed (%)	2.2	1.8
Ramachandran outliers (%)	0	0
Clashscore	4.7	2.8
Average B-factor	56.8	36.8
Macromolecules	56.9	36.4
Ligands	59.8	32.4
Solvent	51.2	42.2

**Table 2:** Interactions between protein and cofactor and/or substrate

Ring	Wt-E_holo_	Interaction	Ring	R173W-ES_2_	Wt-ES_2_ (56MR)	Interaction	Mutations	
			C1	P101	P101	Pyrrole: π-stack	–	
				T102	T102	Ac: H-bond	–	
				V215	V215	Pr: H-bond (main chain)	V215E/M	(Bustad et al., 2013; Schneider-Yin et al., 2008)
			C2	S75^A^	–	Ac: vdW		
				K74^A^	–	Ac: H-bond	–	
				–	F77^B^	Pyrrole: π-stack	–	
				K98	K98	N: H-bond (main chain)	K→R	(Kauppinen et al., 1995)
				D99	D99^A^	Pyrrole: vdW	D→G/H/N	(Floderus et al., 2002; Kauppinen and von und zu Fraunberg, 2002)
				S262	S262	Pr: H-bond	–	
					S262^B^	Ac: H-bond	–	
C1	K98	Ac: H-bond	S1	K98	K98	Ac: H-bond	K→R	(Kauppinen et al., 1995)
	D99	N: H-bond		D99	D99	N: H-bond	D→G/H/N	(Floderus et al., 2002; Kauppinen and von und zu Fraunberg, 2002)
	S146	Pr: vdW		–	S146	Pr: vdW	–	
	S147 S147	Ac: H-bond Pr: H-bond (main chain)		S147	S147	Ac: H-bond	S→P	(Whatley et al., 2009)
	R149	Ac: Salt bridge		R149	R149	Ac: Salt bridge	R→L/P/Q	(Delfau et al., 1991; Gu et al., 1994; Kauppinen et al., 1995; Yang et al., 2008)
	R150	Pr: Salt bridge		–	–			
	R173	Pr: Salt bridge		–	R173	Pr: Salt bridge	R→G/Q/W	(Delfau et al., 1990; Kauppinen et al., 1992; Mendez et al., 2009)
C2	S96	Pr: H-bond	S2	S96	S96	Pr: H bond	S→F	(Kauppinen and von und zu Fraunberg, 2002)
	K98	Ac: H-bond		K98	K98	Ac: H-bond	K→R	(Kauppinen et al., 1995)
	D99	N: H-bond		–	D99^A^	N: H-bond	D→G/H/N	(Floderus et al., 2002; Kauppinen and von und zu Fraunberg, 2002)
	R150	Pr: Salt bridge		R150	R150	Ac: Salt bridge	–	
	A189	Ac: H-bond (main chain)		A189	A189	Ac: H bond (main chain)	–	
	R195	Pr: Salt bridge		R195	R195	Pr: salt bridge	R→C/H	(Kauppinen et al., 1995; Whatley et al., 2009)
	Q217	Pr: vdW		–	Q217^B^	Pr: vdW	Q→H/L/R	(Kuo et al., 2011; Puy et al., 1997; Schneider-Yin et al., 2000, 2006)
				G218	G218	Pr: H bond (main chain)	G→R	(Yang et al., 2008)

^A/B^Refers to the subunits of the asymmetric unit in the crystal structure.

The active-site loop Ser57–Lys74 in R173W-ES_2_ has also been observed in the E_holo_ structure of PBGD from *A. thaliana* (AtPBGD; PDB: 4HTG) and in human wt-hPBGD recently crystallized in the ES_2_-state (wt-ES_2_; PDB: 5M6R). The loop appears to close the active site like a lid, and is mostly unstructured in AtPBGD, whereas it includes a more defined α-helix in both R173W-ES_2_ (Figure 3B) and wt-ES_2_ (PDB: 5M6R). In wt-ES_2_ (PDB: 5M6R), the α-helix is three residues longer and the loop partially covers the active site, without direct interaction with the cofactor or substrates (Pluta et al., 2018). In R173W-ES_2_ presented here, the active-site loop is oriented in a significantly different conformation, partially facing away from the surface with Lys74 establishing several interactions with C2 and S1 (Figures 5 and 6). In wt-ES_2_ (PDB: 5M6R), the pyrrole rings present a similar conformation as in R173W-ES_2_. However, in the mutant structure, we observe a different conformation of the propionate side chain of C1 than in wt-ES_2_ (PDB: 5M6R) (Figure 5), whereas this propionate forms an electrostatic and hydrogen-bonding interaction network with Arg173 and Ser147, the mutation R173W abolishes the interaction and in the crystal structure the propionate is bent and is no longer in contact with the protein (Table 2 and Figure 5).

## Discussion

Despite more than 500 AIP-associated *HMBS* variants discovered to date, little is known about their structural effects. Using high-resolution ESI FT-ICR MS and X-ray crystallography, we obtained crucial structural information on two common AIP-associated mutations, R167W and R173W. Our results are an important step toward understanding the pathogenic molecular mechanisms leading to AIP, as well as the pyrrole-chain elongation-mechanism.

ESI FT-ICR MS allowed us to determine the distribution of the enzyme-intermediates in wt-hPBGD in a quantitative manner, which has not been possible by other methods due to their limited resolution. The ES_2_ intermediate was the most abundant, whereas ES was only found in a very small amount and ES_4_ was not detected at all. ES is kinetically less stable than ES_2_ or ES_3_, and ES_2_ accumulates during the reaction, in agreement with a slow rate of the ES_2_→ES_3_ step (Niemann et al., 1994; Warren and Jordan, 1988). The presence of the apoenzyme in wt-hPBGD is remarkable and has not been observed before in any enzyme preparation is from prokaryote expression, as it is assumed to be unstable and less structurally compact than E_holo_ (Awan et al., 1997; Scott et al., 1989).

Several arginine residues are conserved in PBGD across species. They are involved in or even crucial for either catalysis (e.g., Arg26), structural stability (e.g., Arg251) or implicated in the cofactor binding (Arg150) (Jordan and Woodcock, 1991; Lander et al., 1991). Arg167 is one of the highly conserved residues and has been proposed to act as a gatekeeper for incoming substrates and to be important in breaking of the salt bridges in PBG prior to catalysis (Brownlie et al., 1994; Bung et al., 2018; Gill et al., 2009; Shoolingin-Jordan et al., 2003). Missense mutations of Arg167 affect both the affinity for PBG and the catalytic efficiency of the enzyme rather than instability or misfolding (Bustad et al., 2013; Gill et al., 2009). The proposed effect of Arg167 mutations has recently been attributed to the alteration of the binding site leading to both decreased pyrrole chain elongation and blocking of the HMB release (Bung et al., 2018). Our results clearly demonstrate that the elongation process is indeed perturbed in the R167W mutant. This is consistent with the ∼30-fold higher *K*_m_ and reduced *V*_max_ relative to the wt enzyme (Bustad et al., 2013), which results in slower elongation and accumulation of the reaction intermediates (Bung et al., 2018; Gill et al., 2009; Jordan and Woodcock, 1991; Shoolingin-Jordan et al., 2003). Our results thus do not support the participation of Arg167 solely in product release, since the distribution of enzyme intermediates was rather similar to the wt enzyme, and ES_4_ was not detected at all.

Arg173 is also highly conserved and is considered important for substrate docking to the active site (Louie et al., 1996). The substitution of Arg173 with tryptophan introduces a large hydrophobic amino acid, predicted to hinder the cofactor and/or substrate interaction severely (Jordan and Woodcock, 1991). Both ESI FT-ICR MS and X-ray crystallography show that this mutant accumulates the ES_2_ intermediate with a greatly reduced turnover. Although Arg173 forms hydrogen bonds with the propionate side chains of C1 in the E_holo_ and of S1 in the ES_2_ state (Table 2 and Figure 6) (Pluta et al., 2018; Song et al., 2009), the tryptophan substitution does not hinder the binding of the cofactor and the reaction of the first two PBG substrates. Hence, Arg173 is important for docking the third PBG substrate to the growing pyrrole chain.

Wt-hPBGD is very thermostable with a *T*_m_ of ∼74°C (Bustad et al., 2013, 2020). As seen from our wt-E_holo_ crystal structure and the reported wt-ES_2_ (PDB: 5M6R), this can be attributed to the strong hydrogen-bonding network, in which the cofactor in E_holo_ or the pyrrole chain in ES_2_ engage Arg173 and interacting residues in domain 2, including the cofactor-binding loop Leu257–Val263 in domain 1, with Cys261. In wt-hPBGD, Arg173 might interact directly with S2 and the entering S3 through salt bridges with the acetate and propionate side chains, substituting the interaction of Arg150 with S2 upon ES_3_ formation. The Arg-to-Trp mutation results in a disruption of these interactions, partially explaining the large reduction of thermal stability (Bustad et al., 2013). The conformational change seen in subunit B of the R173W-ES_2_ structure, where the bulky tryptophan residue turns away from the active site forcing a change in the domain structure, probably also affects the loss in thermal stability (Figure S4). Together with its important catalytic function, the role of Arg173 as a stabilizer in the active-site hydrogen-bonding network correlates with the loss of the stability and activity upon Arg-to-Trp mutation and thus a severe AIP outcome.

A striking structural feature of R173W-ES_2_ compared to wt-E_holo_ is that the cofactor-binding loop rearranges to make more space for the two incoming PBG substrates. Furthermore, S1 and S2 in ES_2_ occupy *exactly* the same positions as C1 and C2 of the cofactor in E_holo_ with nearly identical interactions (Table 2 and Figure 6). Despite the alteration of the interactions involving Arg173, the loop conformation and the pyrrole ring locations in our R173W-ES_2_ structure correspond to the structure of wt-ES_2_ (5M6R; Figure 4B), strongly indicating a correct conformation of the intermediate in the mutant. Thus, Arg173 participates in the interaction network involved in the cofactor (C1) and the substrate (S1) binding in the E_holo_ and ES_2_ states, respectively (Table 2 and Figures 5 and 6) (Pluta et al., 2018), but is not important to define the proper conformation of the pyrrole chain. These data corroborate the findings from ESI FT-ICR MS, indicating that Arg173 has a crucial function in the third elongation step from ES_2_ to ES_3_. The catalytic defect is a major consequence of the R173W mutation, despite having mostly been associated with a conformationally unstable protein and concomitantly reduced activity (Bustad et al., 2013; Mustajoki and Desnick, 1985). Its role has, however, not been clearly elucidated in the previous investigations (Bung et al., 2018; Pluta et al., 2018), but its relevance in the interaction network in AIP pathology has been discussed recently (Fu et al., 2019). Nevertheless, our results imply that Arg173 is essential for orienting the intermediate to a specific conformation, in either correctly positioning the pyrrole chain or docking an incoming substrate, allowing the elongation from ES_2_ to ES_3_.

Recent *in silico* investigation of the interaction network during different intermediate states indicates dynamic movement of the active-site loop, as well as specific interactions between the loop and cofactor during the elongation (Chakrabarty et al., 2020). However, the authors do not discuss the rearrangement of the cofactor-binding loop and how the interaction network is affected by this. The active-site loop (Ser57–Lys74) in R173W-ES_2_ adopts a different conformation than in the other crystal structures with electron density describing these residues, i.e., AtPBGD (PDB: 4HTG) or wt-ES_2_ (PDB: 5M6R). In contrast to wt-ES_2_ (PDB: 5M6R), only a short helical turn (Lys62–Thr66) is present in R173W-ES_2_, resulting in a more open conformation including only one clear interaction between the loop and the substrate pyrroles, and Lys74 is hydrogen bonded to the acetate side chain of the C2 pyrrole ring.

Based on the crystal structure of the wt-ES_2_ (PDB: 5M6R), Pluta et al. proposed a mechanism for the reaction progression relying on the further movement on the cofactor-binding loop in the formation of ES_3_ and beyond (Pluta et al., 2018). However, this movement might cause steric issues and major rearrangement of the α3_3_ helix as well as a disturbance of the network of hydrophobic interactions around this helix (Figure S5). Pluta et al. also proposed that Arg26 and Asp99 are the only residues responsible for the pyrrole ring condensations as well as for the release of the product, consistent with the recent computational work by Bung et al. (Bung et al., 2014). The effect caused by the R173W mutantion fits with this mechanism; however, we demonstrate by using this AIP associate mutant rather than the wt enzyme that the substrate elongation from ES_2_ to ES_3_ is crucially dependent on Arg173. Although the Arg-to-Trp substitution does not seem to largely affect the structure of hPBGD in the ES_2_-state, it affects the enzyme stability and the polypyrrole elongation beyond ES_2_, most probably due to the disruption of the Arg173-centered interactions in domains 1 and 2 (Jordan and Woodcock, 1991; Lander et al., 1991).

Understanding the details for the exact elongation mechanism remains unsolved. MD simulations propose a mechanism relying on the protonation of incoming PBGs by the Arg26, and electrophilic addition and deprotonation in concert with Asp99 (Bung et al., 2018, 2019). However, these studies have not considered the movement of the cofactor-binding loop upon the elongation; instead, they are relying on direct helicoidal elongation with only the active-site loop moving. This causes a steric problem for Arg26 and Asp99 where these residues no longer are correctly positioned for the full catalysis cycle. The available crystal structures do not provide detailed information on the dynamic movement of the active-site loop beyond the ES_2_ state, but the wt-ES_2_ (PDB: 5M6R) and R173W-ES_2_ structures clearly show the movement of the cofactor-binding loop.

In conclusion, we show for the first time the direct effect of a mutation associated with AIP on the structure and function of PBGD, revealing the importance of the interaction network around Arg173. Using X-ray crystallography, we trapped the disease-causing mutant R173W in a reaction intermediate and combined with ESI-FT-ICR MS we pinpoint the crucial responsibility of Arg173 in the catalytic mechanism in stabilizing the structure and ensuring proper interaction with the entering substrate to form ES_3_. Furthermore, our work highlights the strength of ESI-FT-ICR MS as a high-resolution technique that quantifies co-existing ES_n_ intermediates, representing an effective procedure to elucidate the catalytic effect of the AIP mutations. Hence, the distribution of the intermediates for the R167W and R173W mutants agrees with the severity of the respective associated AIP phenotype and provides important insights into the catalytic mechanism of PBGD. We propose that as high-resolution MS allows the direct analysis of intermediate distribution in PBGD, it could also be effective for drug screening of e.g., pharmacological chaperones (Bustad et al., 2020), aiming at the stabilization of PBGD and unstable AIP-associated mutants and/or the specific correction of altered distributions of enzyme intermediates.

### Limitations of the study

This study characterized only two AIP-associated mutants. Investigating additional mutants will increase our knowledge on genotype-phenotype relationships and will aid to elucidate the polypyrrole elongation mechanism. The crystallization of the wt-PBGD was performed from a mixture of intermediates, and the heterogeneity of the sample, as well as the flexible parts of the enzyme, such as the N-terminus and the loop including residues 57–74, most likely affect the quality of the data. In the future, this could possibly be overcome by crystallizing isolated intermediates. In addition, a crystal structure of PBGD in the ES_3_-state and/or ES_4_-state is essential for understanding the complete mechanism.

### Resource availability

#### Lead contact

Further information and requests for resources and reagents should be directed to and will be fulfilled by the lead contact, Aurora Martinez (aurora.martinez@uib.no).

#### Materials availability

This study did not generate new unique reagents.

#### Data and code availability

The accession numbers for the protein crystal structure reported in this paper are PDB: 7AAJ, 7AAK. Original data have been deposited to RCSB Protein Data Bank at (https://www.rcsb.org).

## Methods

All methods can be found in the accompanying Transparent Methods supplemental file.

## Supplemental information

Document S1. Transparent methods, Figures S1–S5, and Tables S1 and S2
