# Supplementary material for: Characterization of porphobilinogen deaminase mutants reveals that arginine-173 is crucial for polypyrrole elongation mechanism
Source: iScience. 2021 Feb 6;24(3):102152. doi: 10.1016/j.isci.2021.102152 (PMC7907807; doi:10.1016/j.isci.2021.102152)
Supplement: Document S1. Transparent methods, Figures S1–S5, and Tables S1 and S2 [file mmc1.pdf]

iScience, Volume *24*

Supplemental information

Characterization of porphobilinogen deaminase mutants reveals that arginine-173 is crucial for polypyrrole elongation mechanism

Helene J. Bustad, Juha P. Kallio, Mikko Laitaoja, Karen Toska, Inari Kursula, Aurora Martinez, and Janne Jänis

Supplemental Information

Supplemental Figures

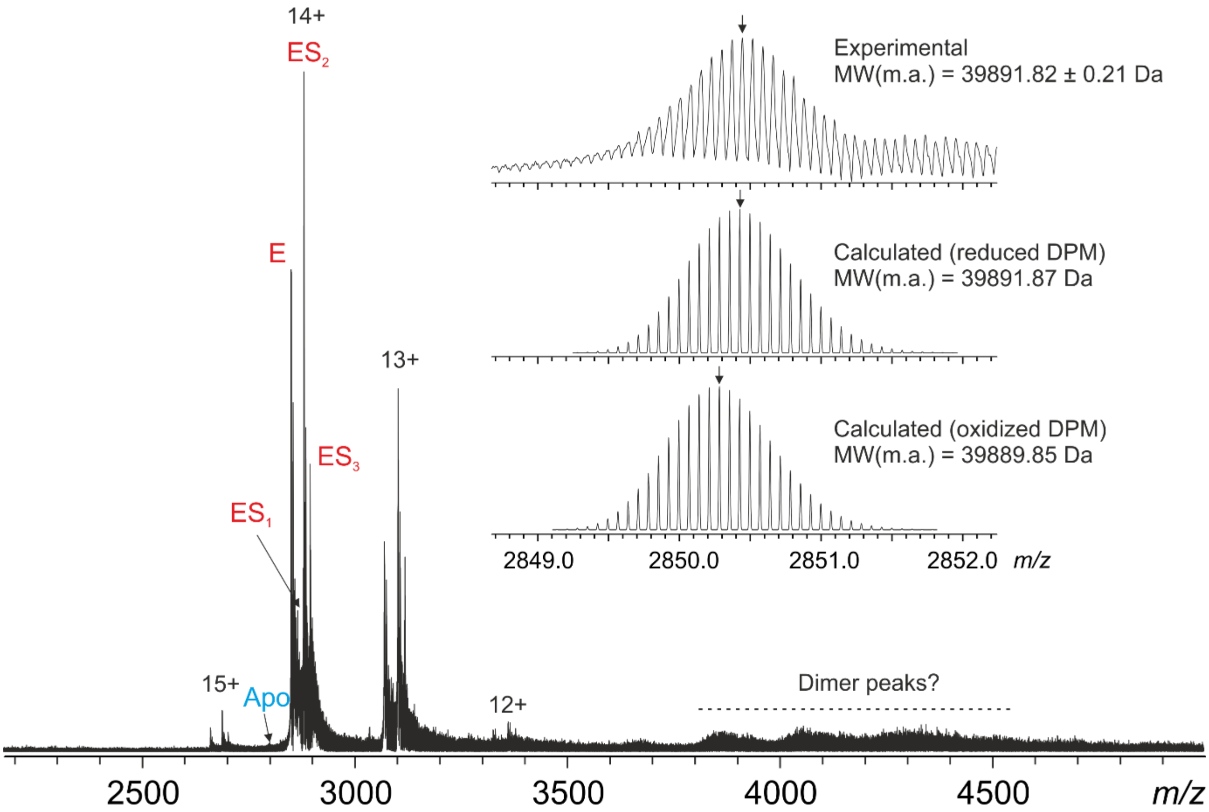

Figure S1. Native mass spectrometry of wt-hPBGD (Related to Figure 2). The ESI FT-ICR mass spectrum was acquired with10 µM protein in 10 mM ammonium acetate pH 6.9. Narrow protein ion-charge state-distribution (12+ to 15+) is consistent with a tightly folded protein conformation. Peaks representing different enzyme-intermediates are assigned for charge state 14+. The inset shows an expanded view on the charge state 14+ of the holoenzyme (E): experimental isotopic pattern (top); calculated isotopic patterns with the reduced (middle) or oxidized (bottom) dipyrromethane (DPM) cofactor present in the structure. The peaks representing the most abundant isotopic masses (MW (m.a.)) are indicated by small arrows. Faint poorly resolved signals at *m*/*z* 3800–4500, possibly representing non-covalent hPBGD dimer are also indicated.

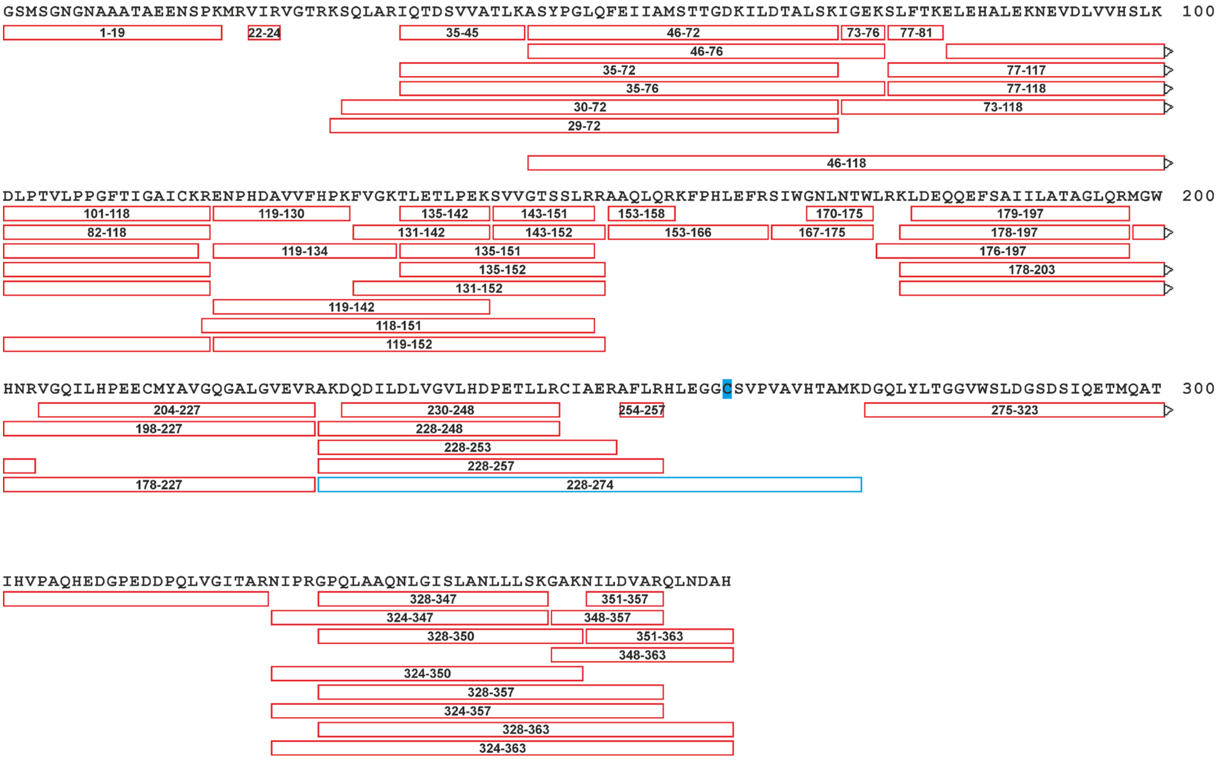

Figure S2. A tryptic peptide map for wt-hPBGD (Related to Figure 2). The numbers in the red boxes indicate corresponding residues for each observed tryptic peptide (note: the numbering here corresponds to the produced protein with two additional N-terminal residues (GS), remnant from the expression vector construct). The blue box indicates the peptide with a covalently modified Cys261 residue (tetrapyrrole = ES_2 intermediate).

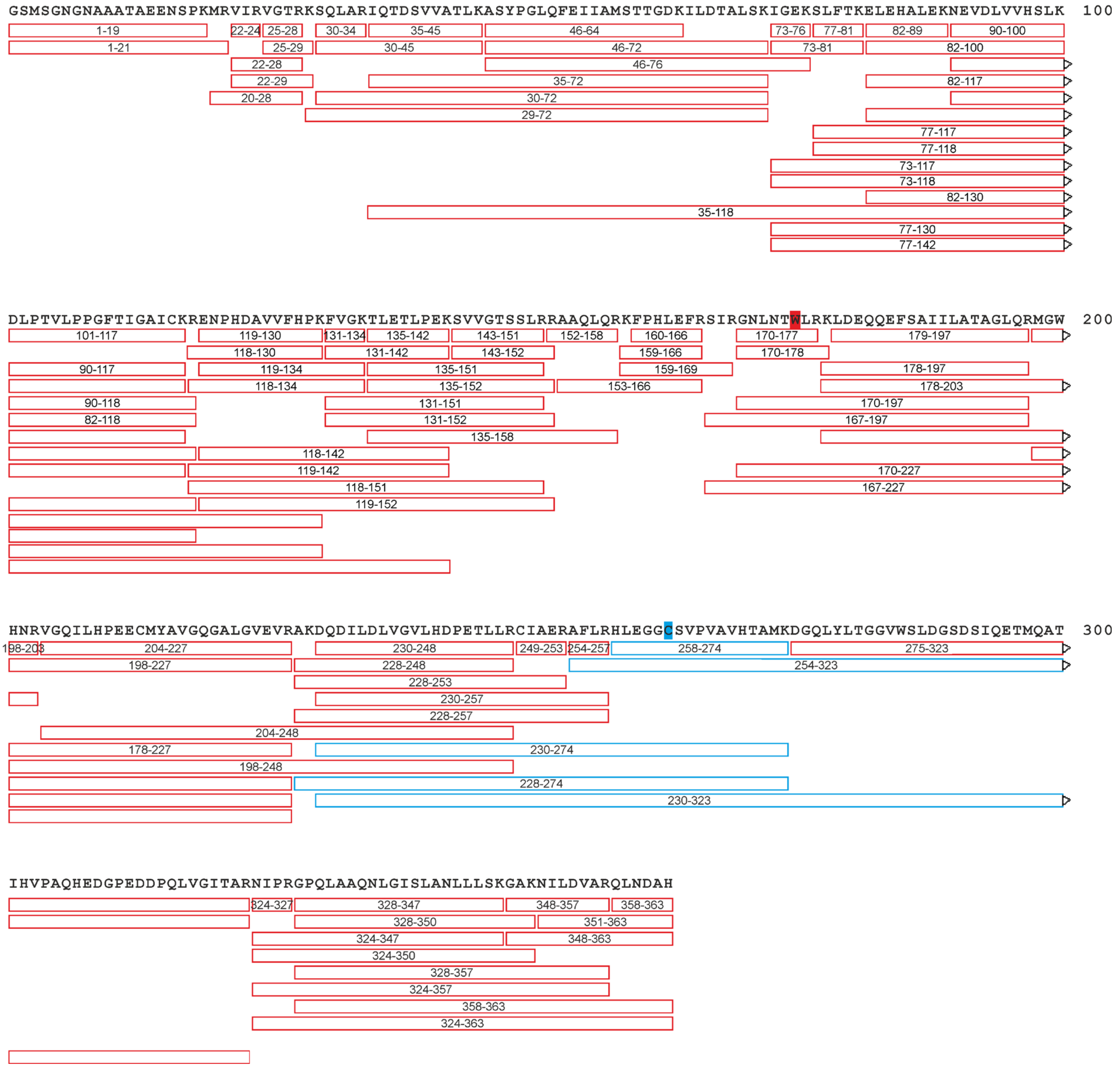

Figure S3. A tryptic peptide map for hPBGD-R173W (Related to Figure 2). The numbers in the red boxes indicate corresponding residues for each observed tryptic peptide (note: the numbering here corresponds to the produced protein with two additional N-terminal residues (GS), remnant from the expression vector construct). The blue box indicates the peptide with a covalently modified Cys261 residue (a tetrapyrrole = ES_2 intermediate).

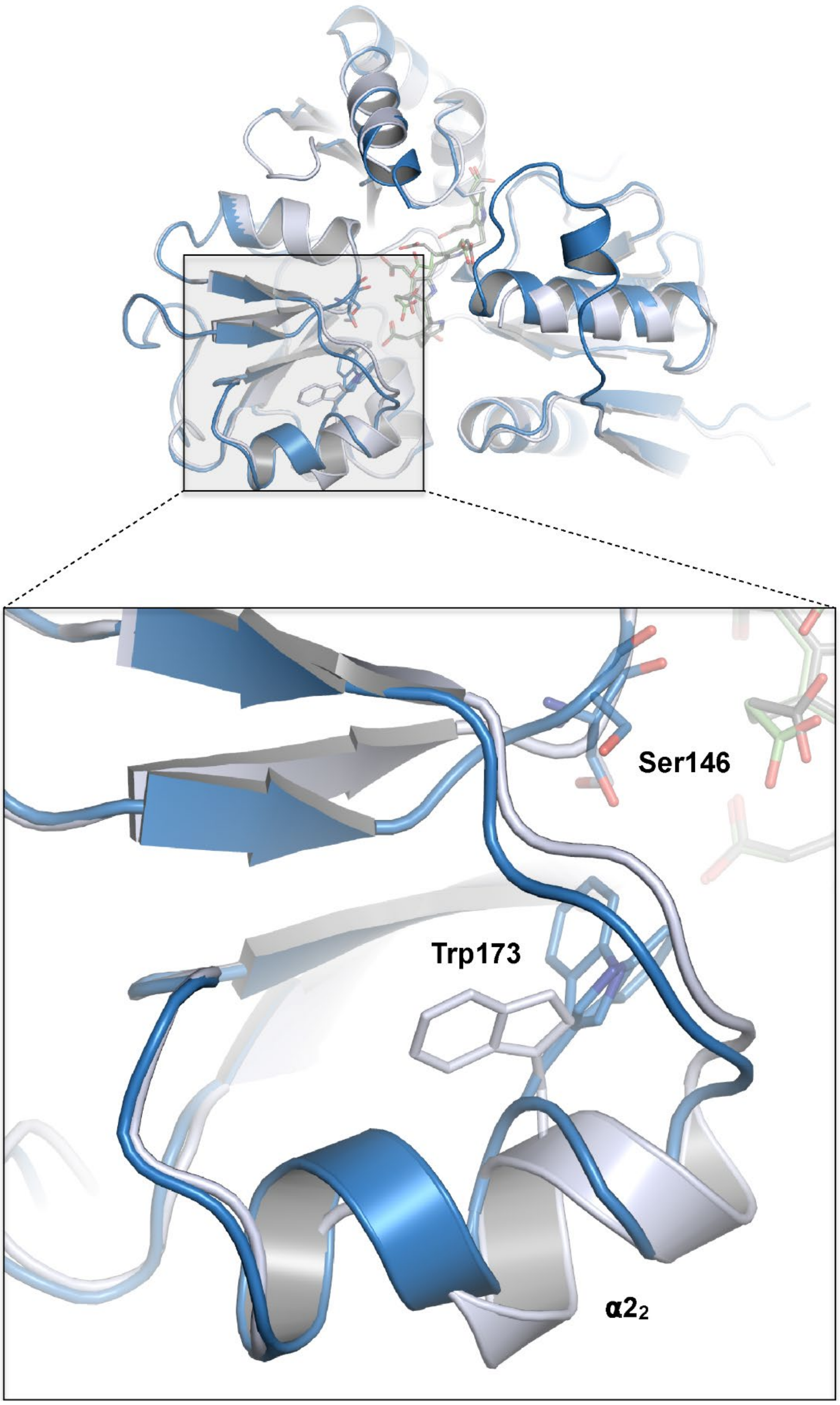

Figure S4. Comparison of the differences between the two molecules in the asymmetric unit in R173W-ES$_2$ (Related to Figure 3). Molecule A (chain A) and molecule B (chain B) are shown in blue and light blue, respectively. Inset for domain 2, with the enlargement below, shows the alternative conformations of Ser146 and Trp173 as well as the rearrangement of in the α2_2 (residues 170–179) in subunit B.

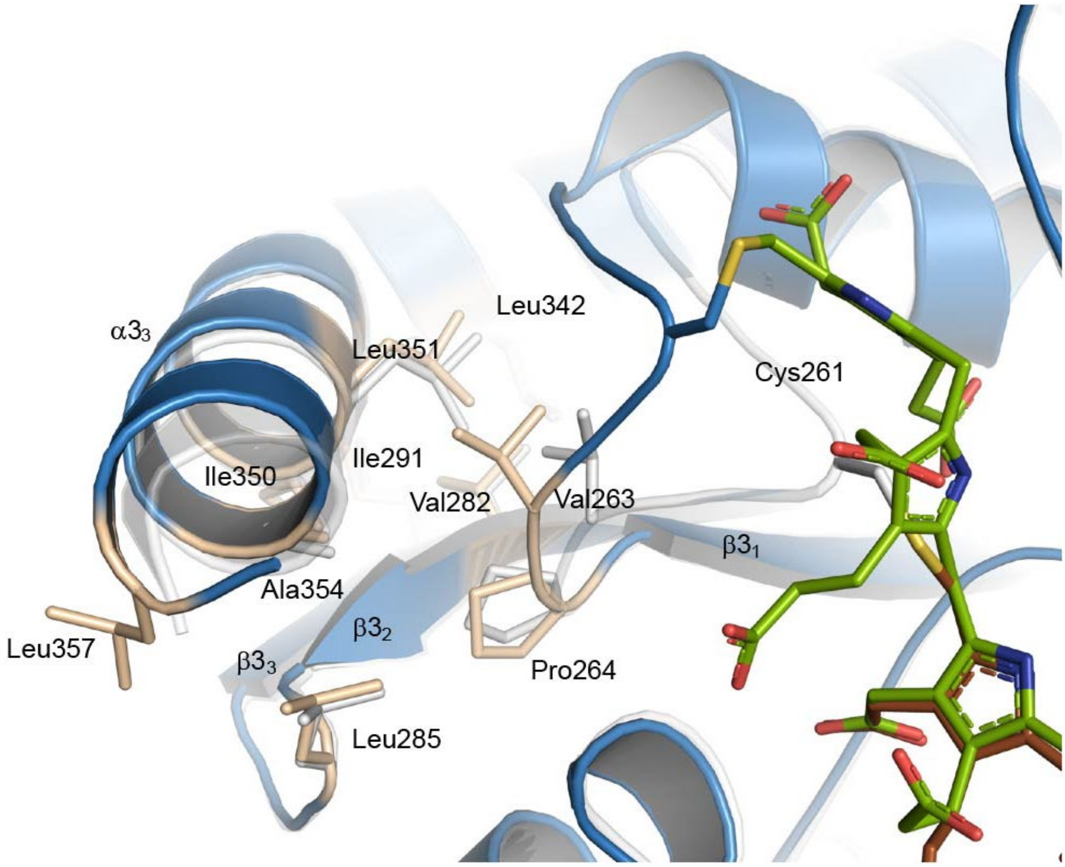

Figure S5. Cartoon representation of mutant R173W-ES$_2$ (blue) and wt-E$_{holo}$ (grey) superimposed showing the hydrophobic interactions anchoring the α3$_3$ in proximity of the cofactor binding loop (Related to Figure 3). Elongation product of R173W-ES$_2$ is shown in green and DPM cofactor with of wt-E$_{holo}$ is shown in brown. Hydrophobic residues on R173W-ES$_2$ between the C-terminal helix (α3$_3$) and the β-sheet (β3$_{1-3}$) are shown in tan. Upon the movement of cofactor-binding loop from E$_{holo}$ to ES$_2$ only small change in Val263 can be detected.

Supplemental Tables

Table S1. List of tryptic peptides observed for wt-hPBGD (Related to Figure 2). The sequence in blue indicates the peptide with a covalently modified Cys261 residue (tetrapyrrole = ES_2 intermediate).

Experimental mass (Da)	Theoretical mass (Da)	Error (ppm)	Residues (start-end)		Sequence
386.26396	386.2642	0	22	24	VIR
445.2543	445.2537	-1	73	76	IGEK
505.30252	505.3013	-2	254	257	AFLR
594.33923	594.3377	-3	77	81	SLFTK
673.35252	673.3507	-3	170	175	GNLNTR
685.3891	685.3871	-3	153	158	AAQLQR
799.45691	799.4552	-2	351	357	NILDVAR
904.49974	904.4978	-2	143	151	SVVGTSSLR
929.5091	929.507	-2	135	142	TLETLPEK
1029.56994	1029.5679	-2	167	175	SIRGNLNTR
1055.61139	1055.6087	-3	348	357	GAKNILDVAR
1060.60153	1060.5989	-2	143	152	SVVGTSSLRR
1173.6635	1173.6605	-3	35	45	IQTDSVVATLK
1360.76376	1360.7602	-3	131	142	FVGKTLETLPEK
1388.68765	1388.6837	-3	119	130	ENPHDAVVFHPK
1477.76755	1477.7637	-3	351	363	NILDVARQLNDAH
1733.92213	1733.9173	-3	348	363	GAKNILDVARQLNDAH
1739.96241	1739.9584	-2	153	166	AAQLQRKFPHLEFR
1791.77445	1791.7694	-3	1	19	GSMSGNGNAAATAEENSPKMR
1815.99992	1815.9942	-3	135	151	TLETLPEKSVVGTSSLR
1819.94175	1819.937	-3	119	134	ENPHDAVVFHPKFVGK
1897.05478	1897.0495	-3	101	118	DLPTVLPPGFTIGAICKR
1972.10014	1972.0953	-2	135	152	TLETLPEKSVVGTSSLRR
2020.17372	2020.1681	-3	328	347	GPQLAAQNLGISLANLLLSK
2102.10677	2102.1008	-3	179	197	LDEQQEFSAIILATAGLQR
2160.14881	2160.1427	-3	230	248	DQDILDLVGVLHDPETLLR
2230.20143	2230.1958	-3	178	197	KLDEQQEFSAIILATAGLQR
2276.32759	2276.3216	-3	328	350	GPQLAAQNLGISLANLLLSKGAK
2359.28078	2359.2747	-3	228	248	AKDQDILDLVGVLHDPETLLR
2403.3552	2403.3486	-3	131	152	FVGKTLETLPEKSVVGTSSLRR
2499.38561	2499.3809	-2	176	197	LRKLDEQQEFSAIILATAGLQR
2500.45497	2500.4489	-2	324	347	NIPRGPQLAAQNLGISLANLLLSK
2554.2737	2554.2672	-3	204	227	VGQILHPEECMYAVGQGALGVEVR
2731.44078	2731.4334	-3	119	142	ENPHDAVVFHPKFVGKTLETLPEK
2756.61002	2756.6025	-3	324	350	NIPRGPQLAAQNLGISLANLLLSKGAK
2869.48535	2869.4783	-2	46	72	ASYPGLQFEIIAMSTTGDKILDTALSK
2931.55694	2931.5488	-3	228	253	AKDQDILDLVGVLHDPETLLRCIAER

3011.54833	3011.54	-3	178	203	KLDEQQEFSAIILATAGLQRMGWHNR
3057.77481	3057.7663	-3	328	357	GPQLAAQNLGISLANLLLSKGAKNILDVAR
3296.73031	3296.7214	-3	46	76	ASYPGLQFEIIAMSTTGDKILDTALSKIGEK
3335.6183	3335.6114	-2	198	227	MGWHNRVGQILHPEECMYAVGQGALGVE VR
3418.84888	3418.8395	-3	228	257	AKDQDILDLVGVLHDPETLLRCIAERAFLR
3538.05541	3538.0471	-2	324	357	NIPRGPQLAAQNLGISLANLLLSKGAKNILD VAR
3736.08447	3736.0748	-3	328	363	GPQLAAQNLGISLANLLLSKGAKNILDVAR QLNDAH
3774.03047	3774.0217	-2	119	152	RENPHDAVVFHPKFVGKTLETLPEKSVVGT SSLR
4025.13911	4025.1283	-3	35	72	IQTDSVVATLKASYPGLQFEIIAMSTTGDKIL DTALSK
4080.21877	4080.2082	-3	82	118	ELEHALEKNEVDLVVHSLKDLPTVLPPGFTI GAICKR
4216.36769	4216.3557	-3	324	363	NIPRGPQLAAQNLGISLANLLLSKGAKNILD VARQLNDAH
4452.38149	4452.3713	-2	35	76	IQTDSVVATLKASYPGLQFEIIAMSTTGDKIL DTALSKIGEK
4500.44618	4500.4342	-3	77	117	SLFTKELEHALEKNEVDLVVHSLKDLPTVL PPGFTIGAICK
4580.45307	4580.4412	-3	30	72	SQLARIQTDSVVATLKASYPGLQFEIIAMST TGDKILDTALSK
4656.54721	4656.5353	-3	77	118	SLFTKELEHALEKNEVDLVVHSLKDLPTVL PPGFTIGAICKR
4708.54925	4708.5361	-3	29	72	KSQLARIQTDSVVATLKASYPGLQFEIIAMS TTGDKILDTALSK
5083.78831	5083.7784	-2	73	118	IGEKSLFTKELEHALEKNEVDLVVHSLKDLP TVLPPGFTIGAICKR
5246.4875	5246.4739	-3	275	323	DGQLYLTGGVWSLDGSDSIQETMQATIHVP AQHEDGPEDDPQLVGITAR
5547.80978	5547.7966	-2	178	227	KLDEQQEFSAIILATAGLQRMGWHNRVGQI LHPEECMYAVGQGALGVEVR
5971.97033	5971.9587	-2	228	274	AKDQDILDLVGVLHDPETLLRCIAERAFLRH LEGGCSVPVAVHTAMK (ES_2)
7935.26651	7935.2462	-3	46	118	ASYPGLQFEIIAMSTTGDKILDTALSKIGEKS LFTKELEHALEKNEVDLVVHSLKDLPTVLP PGFTIGAICKR

Table S2. List of tryptic peptides observed for R173W-hPBGD (Related to Figure 2). The sequences in blue indicate the peptide with a covalently modified Cys261 residue (tetrapyrrole = ES_2 intermediate).

Experimental mass (Da)	Theoretical mass (Da)	Error (ppm)	Residues (start-end)		Sequence
386.2641	386.2642	0	22	24	VIR
431.2502	431.2492	2	25	28	VGTR
445.2550	445.2537	3	73	76	IGEK
449.2653	449.2638	3	131	134	FVGK
498.2932	498.2914	4	324	327	NIPR
505.3034	505.3013	4	254	257	AFLR
559.3457	559.3442	3	25	29	VGTRK
573.3254	573.3235	3	30	34	SQLAR
590.2862	590.2846	3	249	253	CIAER
594.3396	594.3377	3	77	81	SLFTK
696.3211	696.3191	3	358	363	QLNDAH
799.3582	799.3548	4	198	203	MGWHNR
799.5046	799.5028	2	22	28	VIRVGTR
841.4910	841.4882	3	152	158	RAAQLQR
904.5002	904.4978	3	143	151	SVVGTSSLR
927.6016	927.5978	4	22	29	VIRVGTRK
929.5067	929.5070	0	135	142	TLETLPEK
944.4900	944.4868	3	160	166	FPHLEFR
967.5006	967.4975	3	82	89	ELEHALEK
972.5154	972.5141	1	170	177	GNLNTWLR
1021.5833	1021.5808	2	73	81	IGEKSLFTK
1055.6128	1055.6087	4	348	357	GAKNILDVAR
1060.6028	1060.5989	4	143	152	SVVGTSSLRR
1072.5847	1072.5818	3	159	166	KFPHLEFR
1086.6480	1086.6444	3	20	28	MRVIRVGTR
1100.6130	1100.6091	4	170	178	GNLNTWLRK
1173.6598	1173.6605	-1	35	45	IQTDSVVATLK
1251.6853	1251.6823	2	90	100	NEVDLVVHSLK
1360.7645	1360.7602	3	131	142	FVGKTLETLPEK
1388.6879	1388.6837	3	119	130	ENPHDAVVFHPK
1428.8047	1428.7990	4	159	169	KFPHLEFRSIR
1477.7664	1477.7637	2	351	363	NILDVARQLNDAH
1544.7885	1544.7848	2	118	130	RENPHDAVVFHPK
1728.9760	1728.9734	2	30	45	SQLARIQTDSVVATLK
1733.9201	1733.9173	2	348	363	GAKNILDVARQLNDAH
1739.9624	1739.9584	2	153	166	AAQLQRKFPHLEFR
1740.9509	1740.9484	1	101	117	DLPTVLPPGFTIGAICK
1791.7720	1791.7694	1	1	19	GSMSGNGNAAATAEENSPK
1815.9974	1815.9942	2	135	151	TLETLPEKSVVGTSSLR

1819.9433	1819.9370	3	119	134	ENPHDAVVFHPKFVGK
1972.0989	1972.0953	2	135	152	TLETLPEKSVVGTSSLRR
1976.0431	1976.0381	3	118	134	RENPHDAVVFHPKFVGK
2020.1705	2020.1681	1	328	347	GPQLAAQNLGISLANLLLSK
2027.9875	2027.9874	0	46	64	ASYPGLQFEIIAMSTTGDK
2078.9126	2078.9110	1	1	21	GSMSGNGNAAATAEENSPKMR
2102.1069	2102.1008	3	179	197	LDEQQEFSAIILATAGLQR
2160.1414	2160.1427	-1	230	248	DQDILDLVGVLHDPETLLR
2201.1730	2201.1692	2	82	100	ELEHALEKNEVDLVVHSLK
2230.1973	2230.1958	1	178	197	KLDEQQEFSAIILATAGLQR
2247.2525	2247.2475	2	131	151	FVGKTLETLPEKSVVGTSSLR
2276.3270	2276.3216	2	328	350	GPQLAAQNLGISLANLLLSKGAK
2359.2785	2359.2747	2	228	248	AKDQDILDLVGVLHDPETLLR
2403.3518	2403.3486	1	131	152	FVGKTLETLPEKSVVGTSSLRR
2500.4516	2500.4489	1	324	347	NIPRGPQLAAQNLGISLANLLLSK
2554.2674	2554.2672	0	204	227	VGQILHPEECMYAVGQGALGVEVR
2571.1231	2571.1298	-3	258	274	HLEGGCSVPVAVHTAMK (ES_2)
2639.4745	2639.4719	1	135	158	TLETLPEKSVVGTSSLRRAAQLQR
2731.4361	2731.4334	1	119	142	ENPHDAVVFHPKFVGKTLETLPEK
2756.6053	2756.6025	1	324	350	NIPRGPQLAAQNLGISLANLLLSKGAK
2869.4780	2869.4783	0	46	72	ASYPGLQFEIIAMSTTGDKILDTALSK
2887.5403	2887.5345	2	118	142	RENPHDAVVFHPKFVGKTLETLPEK
2931.5500	2931.5488	0	228	253	AKDQDILDLVGVLHDPETLLRCIAER
2974.6231	2974.6202	1	90	117	NEVDLVVHSLKDLPTVLPPGFTIGAICK
3011.5415	3011.5400	0	178	203	KLDEQQEFSAIILATAGLQRMGWHNR
3057.7675	3057.7663	0	328	357	GPQLAAQNLGISLANLLLSKGAKNILDVAR
3130.7239	3130.7213	1	90	118	NEVDLVVHSLKDLPTVLPPGFTIGAICKR
3184.6994	3184.6993	0	170	197	GNLNTWLRKLDEQQEFSAIILATAGLQR
3219.7042	3219.7074	-1	230	257	DQDILDLVGVLHDPETLLRCIAERAFLR
3296.7213	3296.7214	0	46	76	ASYPGLQFEIIAMSTTGDKILDTALSKIGEK
3335.6085	3335.6114	-1	198	227	MGWHNRVGQILHPEECMYAVGQGALGVEVR
3418.8392	3418.8395	0	228	257	AKDQDILDLVGVLHDPETLLRCIAERAFLR
3538.0508	3538.0471	1	324	357	NIPRGPQLAAQNLGISLANLLLSKGAKNILDVAR
3540.9120	3540.9165	-1	167	197	SIRGNLNTWLRKLDEQQEFSAIILATAGLQR
3736.0670	3736.0748	-2	328	363	GPQLAAQNLGISLANLLLSKGAKNILDVARQLNDAH
3774.0233	3774.0217	0	118	151	RENPHDAVVFHPKFVGKTLETLPEKSVVGTSSLR
3774.0233	3774.0217	0	119	152	ENPHDAVVFHPKFVGKTLETLPEKSVVGTSSLRR
3924.1041	3924.1071	-1	82	117	ELEHALEKNEVDLVVHSLKDLPTVLPPGFTIGAICK
4025.1273	4025.1283	0	35	72	IQTDSVVATLKASYPGLQFEIIAMSTTGDKILDTALSK
4080.1971	4080.2082	-3	82	118	ELEHALEKNEVDLVVHSLKDLPTVLPPGFTIGAICKR
4216.3556	4216.3557	0	324	363	NIPRGPQLAAQNLGISLANLLLSKGAKNILDVARQLNDAH

4500.4310	4500.4342	-1	77	117	SLFTKELEHALEKNEVDLVVHSLKDLPTVL PPGFTIGAICK
4580.4320	4580.4412	-2	30	72	SQLARIQTDSVVATLKASYPGLQFEIIAMST TGDKILDTALSK
4656.5325	4656.5353	-1	77	118	SLFTKELEHALEKNEVDLVVHSLKDLPTVL PPGFTIGAICKR
4708.5311	4708.5361	-1	29	72	KSQLARIQTDSVVATLKASYPGLQFEIIAMS TTGDKILDTALSK
4895.5283	4895.5314	-1	204	248	VGQILHPEECMYAVGQGALGVEVRAKDQD ILDLVGVLHDPETLLR
4927.6665	4927.6773	-2	73	117	IGEKSLFTKELEHALEKNEVDLVVHSLKDLP TVLPPGFTIGAICK
5083.7747	5083.7784	-1	73	118	IGEKSLFTKELEHALEKNEVDLVVHSLKDLP TVLPPGFTIGAICKR
5246.4611	5246.4739	-2	275	323	DGQLYLTGGVWSLDGSDSIQETMQATIHVP AQHEDGPEDDPQLVGITAR (ES_2)
5450.8746	5450.8813	-1	82	130	ELEHALEKNEVDLVVHSLKDLPTVLPPGFTI GAICKRENPHDAVVFHPK
5547.7773	5547.7966	-3	178	227	KLDEQQEFSAIILATAGLQRMGWHNRVGQI LHPEECMYAVGQGALGVEVR
5676.8489	5676.8756	-5	198	248	MGWHNRVGQILHPEECMYAVGQGALGVE VRAKDQDILDLVGVLHDPETLLR
5772.8026	5772.8266	-4	230	274	DQDILDLVGVLHDPETLLRCIAERAFLRHLE GGCSVPVAVHTAMK (ES_2)
5971.9411	5971.9587	-3	228	274	AKDQDILDLVGVLHDPETLLRCIAERAFLRH LEGGCSVPVAVHTAMK (ES_2)
6027.1990	6027.2085	-2	77	130	SLFTKELEHALEKNEVDLVVHSLKDLPTVL PPGFTIGAICKRENPHDAVVFHPK
6502.2782	6502.3002	-3	170	227	GNLNTWLRKLDEQQEFSAIILATAGLQRMG WHNRVGQILHPEECMYAVGQGALGVEVR
6858.4893	6858.5174	-4	167	227	SIRGNLNTWLRKLDEQQEFSAIILATAGLQR MGWHNRVGQILHPEECMYAVGQGALGVE VR
7369.9376	7369.9581	-3	77	142	SLFTKELEHALEKNEVDLVVHSLKDLPTVL PPGFTIGAICKRENPHDAVVFHPKFVGKTLE TLPEK
8286.8513	8286.8838	-4	254	323	AFLRHLEGGCSVPVAVHTAMKDGQLYLTG GVWSLDGSDSIQETMQATIHVPAQHEDGPE DDPQLVGITAR
9090.8595	9090.8961	-4	35	118	IQTDSVVATLKASYPGLQFEIIAMSTTGDKIL DTALSKIGEKSLFTKELEHALEKNEVDLVV HSLKDLPTVLPPGFTIGAICKR
11001.3389	11001.2900	4	230	323	DQDILDLVGVLHDPETLLRCIAERAFLRHLE GGCSVPVAVHTAMKDGQLYLTGGVWSLD GSDSIQETMQATIHVPAQHEDGPEDDPQLV GITAR (ES_2)

Transparent Methods

Expression and purification of PBGD proteins

Recombinant human wild-type PBGD and the mutants R167W and R173W were expressed in *Escherichia coli* BL21 (DE3)pLysS (Agilent technologies, Santa Clara, CA, USA) as glutathione S-transferase fusion proteins with a thrombin cleavage site (Bustad et al., 2013). The plasmid for protein expression was generously provided by Professor Pavel Martasek and Dana Ulbrichova as a pGEX4T-1-expression vector (Ulbrichova et al., 2006). Overnight 50 ml pre-cultures supplemented with 100 μM ampicillin and 34 μg/ml chloramphenicol were diluted into 950 ml Luria Bertani broth supplemented with the same antibiotics and 2 g/l glucose and grown at 37 °C in a shaking incubator at 200 rpm until OD_{600} ~0.8. Protein expression was induced by adding 1 mM IPTG (isopropyl thio-*β*-D-galactoside) and grown overnight at 28 °C, 200 rpm. Cells were harvested by centrifugation at 4000 *g* and 4 °C for 15 min at, and stored at −80 ºC. Lysis was performed by sonication on ice after resuspension in lysis buffer (PBS; 140 mM NaCl, 2.7 mM KCl, 10 mM Na2HPO4 and 1.8 mM KH2PO4; 1 mM EDTA, 0.5 mM PMSF, 1 mM benzamidine and cOmplete^{TM} Protease Inhibitor tablet (Roche Applied Science, Penzberg, Germany)), pH 7.4) with three 45-second rounds, 20 W output and 9 sec pulses. Soluble protein was obtained by centrifugation at 14 000 *g* for 45 min at 4 °C, and loaded onto a gluthatione-sepharose 4B column (GE Healthcare, Chicago, Illinois, USA) and washed with 10 × CV of PBS containing 1 mM EDTA, followed by 2 × CV of PBS. The fusion protein was cleaved by adding thrombin (200 units/l) and incubating for 2 hrs at 4 °C on rotation, in 50 mM Tris-HCl, pH 8.0, containing 2 mM CaCl2, 1 mM DTT (dithiothreitol) and 150 mM NaCl. The protein were concentrated with Amicon Ultra centrifugal 30 kDa cut-off filters (Merck Millipore, Burlington, MA, USA), followed by size exclusion chromatography using a Superdex 200 HR16/60 column (GE Healthcare) in 20 mM HEPES with 150 mM NaCl, pH 8.2. After cleavage of the fusion protein, two additional amino acid residues (glycine and serine,

which form part of the cutting site to provide the necessary flexibility for efficient cleavage) remain attached at the protein N-terminus. The protein concentration was measured using a NanoDrop® spectrometer (Thermo Fisher Scientific, Waltham, MA, USA) with the appropriate extinction coefficient predicted by the ExPASy ProtParam web server. The average molecular masses of apo- and holo-PBGD and its intermediates were predicted adding these residues to the ExPASy ProtParam web server based on the following wt-sequence (catalytic Cys261 underlined); mutant residues were substituted where applicable:

```
(GS) MSGNGNAAATAEENSPKMRVIRVGTRKSQLARIQTDSVVATLKASYPGLQFEIIAMSTTG
     DKILDTALSKIGEKSLFTKELEHALEKNEVDLVVHSLKDLPTVLPPGFTIGAICKRENPH
     DAVVFHPKFVGKTLETLPEKSVVGTSSLRRAAQLQRKFPHLEFRSIRGNLNTRLRKLDEQ
     QEFSAIILATAGLQRMGWHNRVGQILHPEECMYAVGQGALGVEVRAKDQDILDLVGVLHD
     PETLLRCIAERAFLRHLEGGCSVPVAVHTAMKDGQLYLTGGVWSLDGSDSIQETMQATIH
     VPAQHEDGPEDDPQLVGITARNIPRGPQLAAQNLGISLANLLLSKGAKNILDVARQLNDAH
```

Mass spectrometry

Prior to the MS experiments, all proteins samples were first desalted/buffer exchanged into 10 mM ammonium acetate, pH 6.9, by using Sephadex G-25 M columns (PD-10; GE Healthcare) and concentrated using Vivaspin 5K centrifugal concentrators (GE Healthcare). The final protein concentrations were estimated by absorbance at 280 nm using sequence-derived extinction coefficients.

Trypsin digestion was obtained by incubating an aliquot of the protein stock solution with TCPK-treated sequencing grade trypsin (Promega, Madison, WI, USA) at a 1:15 (w/w) enzyme-to-protein ratio at 37 °C for 4 h. The digestion was quenched by diluting the samples to the desired protein concentration with a solvent mixture of acetonitrile/water/acetic acid (49.5:49.5:1.0, v/v). The same solvent was used for the intact protein analysis in *denaturing*

solution conditions. For the analysis in *near-native* solution conditions, 10 mM ammonium acetate, pH 6.9, was used instead.

All FT-ICR MS experiments were performed on a 12-T Bruker Solarix-XR instrument (Bruker Daltonik GmbH, Bremen, Germany), equipped with an Apollo-II electrospray ionisation (ESI) source and dynamically harmonised ICR cell (Paracell). All samples were directly infused at a flow rate of 1.5 µl/min using a syringe pump. For intact mass analysis, a total of 300 time-domain transients (1 MWord each) were co-added for each spectrum and zero-filled once to obtain final 2 MWord broadband data. This provided spectral resolution of roughly 200,000 (FWHM) at *m/z* 800 providing baseline-resolved protein signals. The final mass spectra were externally calibrated with respect to the ions of ESI-L Tuning Mix (Part no. G1969-85000; Agilent technologies). The instrument was operated, and the data were acquired with the use of solarixControl 2.0 software and the data were further processed and analysed with Bruker DataAnalysis 4.2 software. Spectral deconvolution for intact protein masses (reported as the most abundant isotopic masses throughout) was accomplished with a maximum entropy deconvolution (MaxEnt), while peptide monoisotopic masses were observed by using a SNAP2 peak-picking module. Sequence analysis (mass calculations) and tryptic peptide identifications were performed with GPMAW 10.0 software (Lighthouse Data). Briefly, monoisotopic peptide masses were matched against the protein sequence within a maximum mass error of 5 ppm with the covalently attached cofactor (DPM) or the polypyrrole as the variable modification.

X-ray crystallography

Both wt-hPBGD and R173W were concentrated to desired concentrations using Amicon Ultra centrifugal filters with 30 kDa cut-off (GE Healthcare) and filtered with 0.22 µm filter prior to the screening for the crystallisation conditions. Screening was performed at two initial temperatures, +8 and +20 °C, and two concentrations, 10 and 20 mg/ml, for both proteins using

vapour diffusion method in SwissCI SD2 sitting drop plates and commercial screens; PACT premier and JSCG plus for Molecular Dimension, and Crystal Screen light and Peg/Ion from Hampton Research. Pipetting was done using Mosquito LCP crystallisation robot (TTP Labtech).

From the initial hits, the conditions were optimised for both proteins. Single crystals were obtained after 4–7 days for both proteins from similar conditions containing 20–25% polyethylene glycol 3350, 200–300 mM ammonium citrate at pH 5.1. The optimised concentrations for wt-PBGD and R173W were 10 and 14 mg/ml, respectively. The crystals for the wt-PBGD were more prone to grow in stacks, whereas R173W crystals were mostly single crystals.

For the data collection, crystals were cryoprotected by soaking for a few seconds in solution having 20% glycerol in addition to crystallisation solution prior to flash freezing. The MX data for the wt-PBGD and R173W was collected at the beamlines P13 and P14, respectively, operated by EMBL Hamburg at the PETRA III storage ring (DESY, Hamburg, Germany). The data was processed with XDS (Kabsch, 2010) and scaled with AIMLESS (Evans and Murshudov, 2013). Phaser-MR (McCoy et al., 2007) from PHENIX suite (Adams et al., 2010) was used for the molecular replacement using PBGD structure 3ECR as a search model. Structures were refined using phenix.refine (Afonine et al., 2012) and validated with MolProbity (Adams et al., 2010). Data collection and refinement statistics are shown in Table 1. All 3D structure diagrams were prepared using Pymol (The PyMOL Molecular Graphics System, Version 2.0.4 Schrödinger, LLC.). The atomic coordinates and structure factor amplitudes have been submitted to the Protein Data Bank (PDB) under the accession codes 7AAJ and 7AAK.

Supplemental References

Adams, P.D., Afonine, P.V., Bunkoczi, G., Chen, V.B., Davis, I.W., Echols, N., Headd, J.J., Hung, L.W., Kapral, G.J., Grosse-Kunstleve, R.W., *et al.* (2010). PHENIX: a comprehensive Python-based system for macromolecular structure solution. Acta Crystallogr. D Biol. Crystallogr. *66*, 213-221.

Afonine, P.V., Grosse-Kunstleve, R.W., Echols, N., Headd, J.J., Moriarty, N.W., Mustyakimov, M., Terwilliger, T.C., Urzhumtsev, A., Zwart, P.H., and Adams, P.D. (2012). Towards automated crystallographic structure refinement with phenix.refine. Acta Crystallogr. D Biol. Crystallogr. *68*, 352-367.

Bustad, H.J., Vorland, M., Ronneseth, E., Sandberg, S., Martinez, A., and Toska, K. (2013). Conformational stability and activity analysis of two hydroxymethylbilane synthase mutants, K132N and V215E, with different phenotypic association with acute intermittent porphyria. Biosci. Rep. *33*, e00056.

Evans, P.R., and Murshudov, G.N. (2013). How good are my data and what is the resolution? Acta Crystallogr. D Biol. Crystallogr. *69*, 1204-1214.

McCoy, A.J., Grosse-Kunstleve, R.W., Adams, P.D., Winn, M.D., Storoni, L.C., and Read, R.J. (2007). Phaser crystallographic software. J. Appl. Crystallogr. *40*, 658-674.

Ulbrichova, D., Flachsova, E., Hrdinka, M., Saligova, J., Bazar, J., Raman, C.S., and Martasek, P. (2006). De novo mutation found in the porphobilinogen deaminase gene in Slovak acute intermittent porphyria patient: Molecular biochemical study. Physiological Research *55*, 145-154.
